# Supplementary material for: Neuronal selectivity to complex vocalization features emerges in the superficial layers of primary auditory cortex
Source: PLoS Biol. 2021 Jun 16;19(6):e3001299. doi: 10.1371/journal.pbio.3001299 (PMC8238193; doi:10.1371/journal.pbio.3001299)
Supplement: S1 Fig — (PDF) [file pbio.3001299.s001.pdf]

Supplementary Information

A complex feature-based representation of vocalizations emerges in the superficial layers of primary auditory cortex

Pilar Montes-Lourido, Manaswini Kar, Stephen V. David, Srivatsun Sadagopan

Supplementary material consists of one supplementary figure and eight supporting data files.

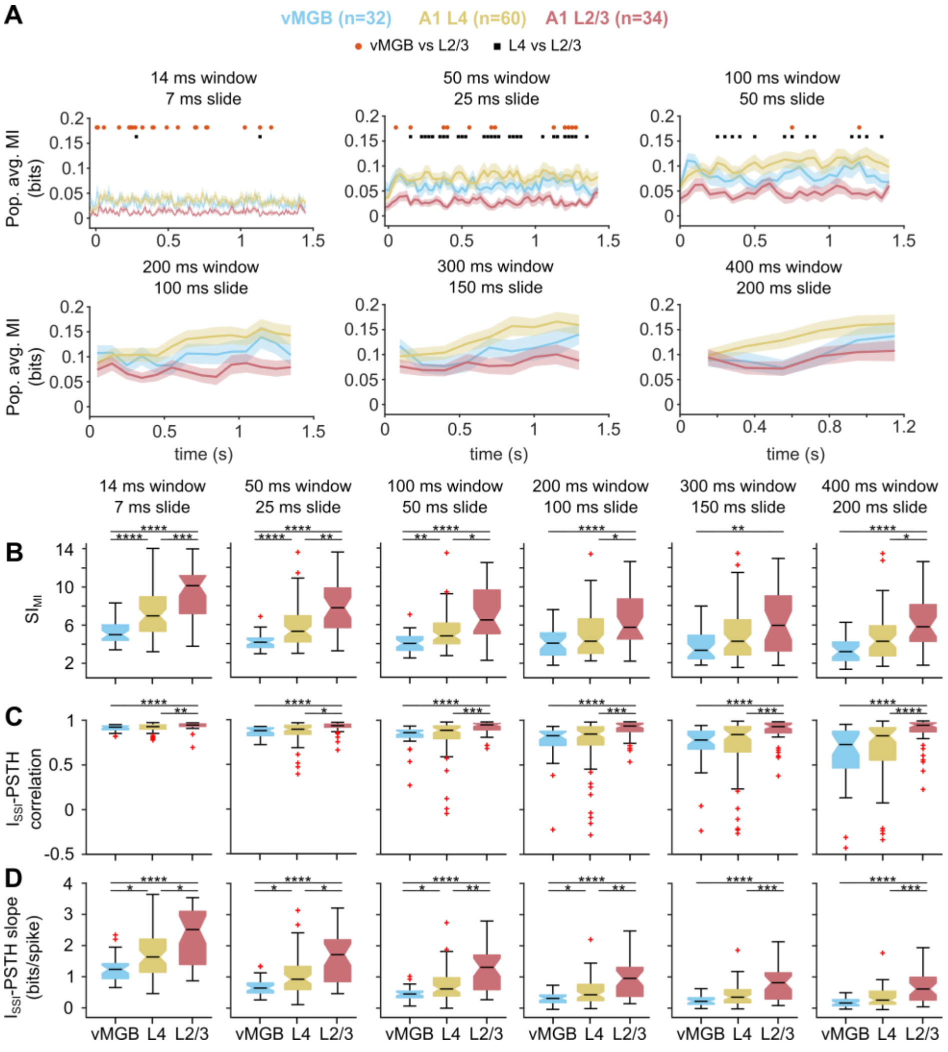

S1 Fig: Mutual information analyses performed across a range of analysis window sizes.

(A) Population average of MI as a function of time in vMGB (blue), A1 L4 (yellow), and A1 L2/3 (red) neurons. Lines correspond to means and shading to 1 s.e.m. Colored dots represent results of statistical testing ($p<0.05$; two-sided t-test with FDR correction for multiple comparisons). Distributions of **(B)** SI_{MI} **(C)** ISSI – PSTH correlation coefficients and **(D)** ISSI – PSTH slopes for vMGB, A1 L4 and A1 L2/3 neurons at all considered window sizes. Asterisks correspond to: *: $p<0.05$, **: $p<0.01$, ***: $p<0.005$, ****: $p<0.001$ (Kruskal-Wallis test with posthoc Dunn-Sidak tests). Data underlying this figure can be found in supporting file S9 Data.
