# Supplementary material for: Neuronal selectivity to complex vocalization features emerges in the superficial layers of primary auditory cortex
Source: PLoS Biol. 2021 Jun 16;19(6):e3001299. doi: 10.1371/journal.pbio.3001299 (PMC8238193; doi:10.1371/journal.pbio.3001299)
Supplement: Attachment — Submitted filename: Response_to_reviewers_Rev2.pdf [file pbio.3001299.s012.pdf]

Response to reviewers:
We thank all the reviewers again for their constructive feedback that has greatly improved our manuscript.

Changes not requested by reviewers:

- We replaced Figure 4 with a revised version, as there was a minor copy and paste error of kurtosis values displayed in panel 4C.

Editorial requests:
1) Ethics request: In your methods section, please include the identification number of your protocol, approved by the University of Pittsburgh IACUC.
- The protocol number is provided in page 28, line 703.

2) Data request: Please provide, as a supplementary file, the underlying data for each figure in your study. Please also reference this dataset in the figure legends. For example, to each figure legend you might add the following statement: “data underlying this figure can be found in supplementary file S1_data.” You will also need to ensure that this data file contains a legend, and is referenced in your data availability statement. I have included more information regarding our data sharing policy and this request below my signature.
- Data underlying figures have been uploaded. Please note that Figure 3 is a spike raster plot which is raw data, consisting of thousands of dots denoting spike times. It is neither feasible nor useful to share the coordinates of each individual dot. The data supporting the analyses of spike times and rates (Figure 4) has of course now been uploaded.

3) Thank you for changing your title in response to reviewer comments in the last round of review. We have been discussing the title of your manuscript, and wonder if it might be edited slightly to be more accessible to a broader readership? If you agree, you might change it to something like 'Neuronal selectivity to specific vocalizations emerges in the superficial layers of primary auditory cortex'

- Thank you for this suggestion – we have amended the title to **“Neuronal selectivity to complex vocalization features emerges in the superficial layers of primary auditory cortex”**. We prefer “vocalization features” to “specific vocalizations” because it more narrowly reflects the results. We added the “complex” qualifier because, as reviewer 1 pointed out in the first round of review, frequency can also be considered a vocalization feature.

4) Please take a moment to review your reference list to ensure that it is complete and correct. If you have cited papers that have been retracted, please include the rationale for doing so in the manuscript text, or remove these references and replace them with relevant current references. Any changes to the reference list should be mentioned in the cover letter that accompanies your revised manuscript.

- We have checked the references and could not find retracted studies – could you please inform us if you have indeed identified such references? We would be happy to delete or justify specific references.

Reviewer #1:
I would like to thank the authors for conducting extensive additional analyses, which confirm the conclusions they drew in the original version of this manuscript. I have no further comments.
Typo in lines 174, 176, 413: ANOCOVA --> ANCOVA

- We have corrected these typos, thank you for bringing them to our attention.

Reviewer #2:

All my comments on the previous version were taken into account, the manuscript was substantially improved and I do not have other comments.

- We thank the reviewer for their time and effort in improving our manuscript.

Reviewer #3:

The manuscript from Montes-Lourido et al is improved and makes a more convincing case for a transformation in the representation of species-specific vocalizations between cortical layers in the guinea pig. The authors are creative in their analyses of the responses and have generally provided transparent and rigorous statistical analyses to illustrate their points. Their new Table nicely summarizes differences between the areas, though statistically speaking, it would make more sense to compute effect sizes and list those rather than the degree of significance in a statistical test.

- We have now included effect sizes in addition to degree of significance in Table 1. Specifically, we used the non-parametric measure of effect size, Cliff's delta. Please see page 21, lines 543-546 for details. We could not find a reference to compute effect size for comparing 2-D matrices, or across multiple time points.

However, one issue that remains a serious concern is the mutual information (MI) analysis, which has been expanded to include data from vMGB in addition to A1 L4 and L2/3. The authors' explanation of having to "fill-in" the spontaneous activity to compute the MI is appreciated, but their full explanation did not make it into the Methods, and that should be updated. It would also be helpful to the reader to know exactly which stimuli received these filled-in spikes (Purr?) since there are examples in Fig. 8F-H that seem to show abrupt drops in SSI about 1 second after the end of the stimulus. Given recent studies of auditory cortical Off responses after the end of stimuli, the act of substituting spiking could potentially skew results. Checking whether neurons exhibit firing rate differences over the last 1 sec vs. the simulated 1 sec of spontaneous activity, and how that affects the calculated MI, would help assuage this concern, but at the least some clearer statement about what was done and its justification in the Methods seems warranted.

- In light of the reviewer's concern about "filling in" spikes, in this revision, we have amended our analysis to not contain any simulated spikes. To accomplish this, we only analyzed the first 1457 ms of responses, and truncated the later responses of longer calls. This truncation length was determined by shortest duration call (the Purr call referred to by the reviewer, 457 ms) plus the 1000 ms post-stimulus period where we did not have interference from following stimuli. This truncation method is based on earlier published mutual information analysis of vocalizations of different lengths (please see Gaucher et al., J. Neurosci. 2013). Our conclusions remain unchanged by this revised analysis. We hope the reviewer appreciates the robustness of this result as it holds across quite different windows of analysis.

- We have now amended Figure 8 and the supplementary Figure to reflect these new analyses, and have amended the main text and methods to describe the truncation. We also provide new p-values. Please see page 18, line 467 and Methods page 35, line 886.

More seriously, although the filling-in explanation now helps me understand what the authors did in computing the MI, I am left somewhat perplexed by how they then use these results to support their conclusions. The authors make a point about longer temporal integration times in L2/3 based on the area-under-the-curve of their bin-by-bin mutual information curves. This seems problematic. The Methods indicate that they use a sliding window with overlap between points, meaning that each time

bin is not independent from each other. Moreover, the MI can obviously be negative because of their shuffle correction subtraction (e.g. Fig 8C), but it is not clear what constitutes the AUC in that case. In general, there is no obvious physical or conceptual meaning to the AUC of their MI curve aside from it being used to show a difference between neural regions. If the authors intended the AUC to essentially represent the average MI over the duration of the sounds, then at the least they should eliminate the non-independent points that overlap in that average. Even still though, computing an average MI for a neuron in this fashion is questionable because of the nonstationarity of the stimulus over the time bins and correlations in firing between time bins. A methodologically more sound way to estimate MI would be to divide time windows into smaller bins and create spike words. It takes many trials to estimate the probability distribution across words though, and it is not clear whether enough trials were run to do this well. Estimating MI based on spike counts (not words) in large windows as done by the authors may be ok (though does not get at "activity patterns" as they claim), but it is still not clear what averaging or summing the values from adjacent windows (as was done to compute the AUC) means.

As far as supporting a point about integration time, Fig. 8B does show that L2/3 looks different as a function of the window length, but there are no error bars, and no explanation of what normalization was used. Thus, it is hard to know what exactly to make of the shift to the right of that curve and why the three curves meet at 400 ms. The authors seem to be arguing that because the AUC for L2/3 neurons does not saturate above 200 ms, the integration time for L2/3 is longer than in the other two brain areas. This argument is based on the rate of change of the AUC with increasing window length, and exactly how that relates to the integration time is unclear. If anything, because the authors are essentially counting spikes in ever larger windows for their MI calculation, the exact timing of the spikes within the window becomes less important as the window size increases, so the L2/3 result seemingly says more about worsening temporal precision rather than how long a stimulus window is needed to drive responses (integration time). However, that interpretation seems to be at odds with the conclusions from Fig 4B, where the authors argue that the feature response window for L2/3 neurons is actually smaller than in L4 and vMGB. Hence, the concern is that the AUC approach to characterizing the MI and how temporally extended "patterns" of firing conveys information is leading to difficult to interpret results, and the authors should reconsider whether to include it.

- In light of the reviewer’s concerns, and as suggested by the reviewer, we have decided to remove the AUC analysis from our manuscript. We have removed old Fig. 8B and the section of main text describing these results

Finally, also in the context of computing information, the authors refer to high-MI points, and highlight these in Fig 8C-E with red crosses. However, the Methods do not explain how these were chosen; they appear to be the peaks, but not all peaks are selected. Since high-MI points were used in their subsequent correlation analysis with the PSTH, it is important to explain this in detail. Are their results changed if they use all bins rather than just those with high MI?

- The high-MI time bins were previously defined in the Methods (now page 36, line 907) as bins that exceeded 1 standard deviation of the MI values across all time bins. We also included a note in the main text in this revision (page 20, line 517).

- The high-MI time bins were only used for the SI_{MI} analysis (Fig. 8H) because, as we explained, we wanted to see whether the high information content in some population MI time bins was distributed across all stimuli or if it was restricted to few stimuli. The PSTH-I_{SSI} correlation and PSTH-I_{SSI} slope analyses in fact used all time bins and not just the high-MI bins.
