# Supplementary material for: Neuronal selectivity to complex vocalization features emerges in the superficial layers of primary auditory cortex
Source: PLoS Biol. 2021 Jun 16;19(6):e3001299. doi: 10.1371/journal.pbio.3001299 (PMC8238193; doi:10.1371/journal.pbio.3001299)
Supplement: Attachment — Submitted filename: Response_to_reviewers_Rev1_v2.docx [file pbio.3001299.s011.docx]

**Response to Reviewers**

We thank the reviewers for their detailed and insightful reviews, and the editor for the opportunity to resubmit this manuscript. We have added substantially to the manuscript, and we are grateful to the reviewers for their efforts in helping us improve the manuscript.

This study was originally submitted as a ‘Discovery Report’, and after editorial review, amended to a ‘Short Report’. Our original intention was to report an initial phenomenon (the emergence of call feature selectivity in the superficial layers of primary auditory cortex), which we could then follow up with further detailed analyses. Indeed, many of the reviewer questions were ‘in the pipeline’ but delayed due to Covid-related complications.

Having read the reviews, however, it is clear that more complete analyses were necessary to support our claims. Therefore, we have now expanded the original manuscript with some additional data as well as extensive new analyses to address all reviewer concerns. We collaborated with Dr. Stephen V. David on receptive field estimation, and have added him as a co-author on the manuscript. These additions have necessitated the inclusion of 5 new main figures as well as expanded main text. We therefore request that rather than being treated as a ‘Short Report’, this revision be treated as a regular research article.

**Reviewer #1:**

1. Abstract: Is "… optimized for complete representations of sounds" the best phrasing? Neurons are also optimized for the complete representation of a sound if they are category selective. Perhaps "neurons faithfully reflect acoustic input"?

- We have changed the wording as suggested (Page 2, Line 32)

2. Abstract: the use of the term feature-selectivity is confusing (4th sentence), as it could be taken to mean simple tonotopic tuning (i.e., frequency is an acoustic feature after all).

- We modified the sentence as “But how neural selectivity for informative call features emerges…” (Page 2, Line 37)

3. Caption of Figure 2: should this say "call-responsive" instead of "call-selective" neurons? The first neuron in A responds to all 8 call categories, so this neuron is not call-selective.

- Yes, “call-responsive” is more accurate. We have modified the caption as suggested. (Figure 3 caption in revised manuscript).

4. The analyses that underlie Figures 3B-C combine two effects: that of call-selectivity and the response duration per call. That is, it does not allow discriminating if regions differ from each other because neurons respond to fewer calls, or respond shorter (or a combination of both). It would be interesting to restrict these analyses to sounds for which there is at least one responsive window.

- We agree and thank the reviewer for this suggestion. The differences in the presence vs. absence of a response window was already captured by our selectivity metric. Therefore, we now present analysis where we have restricted comparisons only to calls where at least one response window was detected (Figure 4B in the revision). Further, to get at the question of whether neurons respond to shorter segments of the call, rather than plot the lengths of individual windows, we now compare the total response duration (sum of the lengths of all response windows). We think that this representation clarifies that whereas vMGB and A1 L4 neurons use several response windows to respond to a greater duration of calls, A1 L2/3 neurons use 1 or 2 windows to respond to a much more restricted duration.

5. Fig. 3D: how can the y-axis (resp. window length [fraction of call length]) go higher than 1? Are there neurons that respond longer than the sound duration?

- Yes, we do observe some responses that persist beyond the stimulus duration. Such post-stimulatory activity has been previously reported in the auditory cortex of awake, passively listening animals (Cooke JE et al., Sci. Rep. 2020).

6. Results, page 11: "These data suggest that vMGB and A1 L4 neurons are likely driven by the frequency content of calls, when call spectral energy overlaps with the neurons' tone receptive fields. Consequently, vMGB and A1 L4 neurons show poor call selectivity because call spectral energy remains more or less consistent over the call duration and is similar across GP call types. In contrast, despite this overlap of spectral energy across call types, A1 L2/3 neurons respond only in narrow windows because they are likely driven by specific spectrotemporal features that occur during calls, consistent with an earlier theoretical model [29]." This is a relevant and interesting conclusion. However, the reason for poor selectivity in MGB/A1 and reason for the narrow response windows in A1 L2/3 is speculation; this is not supported by the data. Is it possible to better support this statement by the data? For example, by estimating RFs (showing the emergence of combination

selectivity in L2/3), and/or by providing more information on the acoustics of the calls and relating that to the neural best frequency + call responsivity?

- We agree that the statements regarding the relationship between tuning properties of neurons and their call responses were not supported by the analyses in the original submission. In the revision, we now provide extensive new analyses, summarized in new Figures 5, 6, and 7, characterizing the receptive fields of call-selective and non-selective neurons across processing stages. Briefly, we used an available software package (NEMS) to fit linear-nonlinear (LN) models to call-responsive neurons. We observed that while the model performed similarly across the processing stages during training, the model did not generalize well to a validation dataset particularly in A1 L2/3, demonstrating the difficulty of capturing these responses using LN models. In addition, we show several examples of frequency-selective receptive fields in L4 and feature-selective receptive fields in A1 L2/3. Critically, we show that STRF complexity is not different between vMGB and A1 L4, but is significantly higher in A1 L2/3. These new analyses support our original statement that while A1 L4 responses are likely driven by spectral content (given the restricted frequency-selectivity of their STRFs), A1 L2/3 neurons are tuned for complex spectrotemporal features. (Please see new Figures 5, 6, and 7; Pages 13 – 18 of main text).

7. Results, page 13: "… could not be attributed to low-level differences in tuning properties". Perhaps rephrase, as it is not clear what is meant by "low-level differences". After all, differences in spectrotemporal tuning may also be termed low-level. Instead, the authors seem to intend to refer here exclusively to frequency tuning.

- Thank you for pointing this out – we have now rephrased the statement as
“… could not simply be attributed to differences in frequency tuning”. (Page 12, Line 306).

8. Figure 4: Why is data from the ventral MGB missing from the MI analyses? How does it compare to A1?

- We now present MI data from the vMGB. We show that the MGB population MI is not significantly different from the L4 population MI. The sparsity of MI showed an increasing trend between MGB, L4 and L2/3. The I_SSI_-PSTH correlation was not significantly different between vMGB and A1 L4. Interestingly, we believe that some of these results can be attributed to the timescales of information processing across the three stages – in new analysis summarized in Fig. 8B, we show that while the total population MI saturates within about 200 ms in vMGB and A1 L4, A1 L2/3 MI does not saturate until at least 400 ms. (Figure 8 of revised manuscript).

9. Discussion: "While vMGB and A1 L4 neurons seemed to respond primarily to the spectral content of calls …" and "A1 L2/3 responses were contingent on the presence of specific spectrotemporal features". This is an interpretation, not a result. Either the analysis needs to be extended to support this statement in the Discussion (see comment 6; for example by showing a non-linearity in the RFs), or the authors should better clarify what is a result and what is interpretation.’

- We agree that in the original manuscript this was indeed an interpretation. However, in the revision, we believe that we have now provided analyses to support this statement (please see response to comment 6 above, Figures 5, 6, and 7, pages 13 – 18).

**Reviewer #2**:

i/ Grimsley et al. (2012) demonstrated in narcotized guinea pigs that "the primary area (AI) and three adjacent auditory belt areas contain many units that give isomorphic responses to vocalizations". According to them "area VRB (ventrolateral belt) has a denser representation of cells that are better at discriminating among calls by using either a rate code or a temporal code than any other area". Although the authors mention the paper by Grimsley et al. (2012) in their manuscript, I believe it deserves more attention in the Discussion. Speculations, how information about the specific features of animal´s vocalizations, as detected in the A1 L2/3 by the authors, will be transmitted and used in the secondary areas of the guinea pig´s auditory cortex will be appreciated.

- While we had briefly described our working model of how highly feature-selective responses could be generated, due to the ‘Short Report’ format we had not discussed further processing steps in our original submission. In the revision, we have included a new Figure 9 and extended the discussion section on our working model for how feature-selective neurons in A1 could lead to the types of responses observed by Grimsley et al. in secondary cortices. (New Figure 9, pages 25 – 26 of revised manuscript).

ii/ Traditionally different types of vocalizations are illustrated by their spectrograms and waveforms. This information is missing in the manuscript, although the results are based on 8 different vocallzation categories. The spectrograms and waveforms of vocalizations used by the authors could be shown in the Supplementary material.

- We thank the reviewer for this suggestion and agree that it is certainly informative to present the stimuli used in these experiments. Given the central importance of the stimuli to the study, as well the point raised by Reviewer 3 regarding the relative scarcity of A1 L2/3 neurons in the 3-6 kHz range, we believe that this figure merits inclusion in the main manuscript. Therefore, spectra and spectrograms of vocalizations are now presented in new Figure 2 in the revised manuscript.

iii/ Fig. 3D shows joint distributions of the number and length of response windows. vMGB and A1 L4 neurons exhibit either multiple short windows or a single long window. In contrast, A1 L2/3 neurons exhibited one or two short response windows. The significant difference between vMGB and A1 L2/3 and between A1 L4 and A1 L2/3 is not surprising but why is there large difference between vMGB and A1 L4? This finding deserves at least some explanation in the Discussion.

- We believe that this was due to an interaction between how we counted the number of occurrences in each cell of the Number of Windows x Window length matrix and the bin size used. For example, in the original submission, if we encountered 5 response windows, say each of 0.18 s duration, we would fill the cell corresponding to 5 windows and 0.1-0.2s length with a count of 5. But if we encountered 5 windows of length 0.2 s, the entire count of 5 would be attributed to the 0.2-0.3 s bin. Thus, although the vMGB and A1 L4 matrices looked similar on the surface, they were significantly different. In the present version, we plot Number of Windows against total fractional response length which is more tolerant to small changes in the bin size. In other words, we can capture how many response windows there were per response, and what the total duration spanned by these windows were. In this representation, vMGB is not significantly different from A1 L4. (please see Figure 4 of the revised manuscript).

iv/ There is some inconsistency in the presented illustrations and descriptions of the call selectivity/responsiveness. Fig. 1G suggests there are about the same portions of neurons responding to individual calls in all structures, i.e. the overall ratio of responsiveness between MGB and A1 L2/3 is about 1:1. In contrast to this Fig. 3A suggests that the responses to calls are less frequent in the A1 L2/3 than in MGB with the ratio about 4.5 : 6.5. It is not easy to understand how the values for Fig. 1G have been calculated (e.g. for 28 MGB neurons should be the blue bars of percentage with the step of 100/28 = 3.6%, but the differences among the blue columns are much lower).

- Suppose a neuron in MGB responds to all 8 calls. Then, for this neuron, we would add a count of 1 to all 8 call categories. We repeat this for all neurons to determine how many times each call has evoked a response in an MGB neuron, and divide by the total number of calls evoking a response, resulting in the plotted probability distributions. We repeat this for the different processing stages. In A1 L2/3 individual neurons may only respond to 2 or 3 categories, but across the population the responses are spread out evenly across all categories. Thus, when the probability distribution is plotted, it is also uniform.
- Perhaps the source of confusion was the y-axis label of Fig. 1H (earlier labeled ‘% Neurons’). We have changed the label to ‘% Responses’ in the revision.

In addition, the Fig. 1G is also used as a proof that the illustrated effect of the emergence of call selectivity in A1 L2/3 is not due to only some of the call types (lines 295-300) " This was not the case in our data - neural preference for call type was evenly distributed across all tested call types across the processing stages (Fisher's exact test [37], p = 0.99; Fig. 1G)."

This statement should be supported by some analysis (like a kind of bootstrap).

- The statistical test we used to support this statement in the original submission was the Fisher’s exact test, which was not significant. We now performed a two-sided permutation test with 1000 iterations to evaluate whether we found differences in the proportion of responses for each call type across layers (8*3 = 24 tests). We performed an FDR correction for multiple comparisons. We found a small over-representation of ‘Other’ calls and a small under-representation of ‘Purrs’ at a significance level of p=0.014. (Please see Figure 1H of the revised manuscript).

v/ It could be also interesting if responses to tone and/or noise stimuli are included into the analysis, to point out if the change in selectivity is specific for complex stimuli (like calls).

- We agree, but unfortunately we did not use the same stimulus set of tones or noise across all the recorded neurons – we typically sampled about 3 or 4 octaves around the neurons best frequency with variable number of steps per octave. Therefore, our present dataset does not lend itself to this analysis. However, this is indeed a very important question that we are presently trying to address in ongoing experiments, including by constructing artificial complex sounds.

vi/ Line 610. How many neurons from individual structures responded to vocalizations by inhibition?

- We encountered 2 such neurons in A1 L2/3 and 9 neurons in A1 L4 (added to Page 33, Line 818).

Minor points:

Line 93. This sentence requires citation.

- We apologize for the misunderstanding; this sentence refers to the theoretical work from our lab that we refer to earlier in the paragraph. The citation has now been added (Page 4, Line 97).

Line 153. Please explain what means LFP responses.

- We have added the expansion, ‘local field potential (LFP)’, to this line (Page 7, Line 161)

Line 513. How about placement of the chamber for electrophysiological recording in the case of MGB?

- As we stated in the original manuscript, used the same chamber but an angled electrode track to reach the MGB (Page 30, Line 753 of revised manuscript).

Line 519. ….at a sampling rate of 100 kHz …

- Corrected here and at other locations.

Fig. 1D. Please explain what means CSD.

- CSD is current source density and is expanded in the main text (Page 7, Line 162). We define that this refers to the second spatial derivative of the LFP in the methods section (Page 31, Line 776).

**Reviewer #3:**

1) The authors provide good transparency on much of their neural data, but there is critically missing information about the spectral content of the vocalizations used in their study and its overlap with the best frequencies of neurons recorded. This is problematic because of potential differences in their L2/3 vs. L4 and vMGB neural populations. Many gerbil vocalizations fall heavily in the 2-10 kHz range, and especially around 3-6 kHz. This is a frequency range where there is a lack of best frequencies in the recorded population of L2/3 neurons, unlike for L4 and vMGB (Fig 1F). Even though similar percentages of neurons across areas respond to the different calls (Fig 1G), if the same types of neurons are not being sampled, then either those BFs don't exist in L2/3 (potentially interesting but unlikely given results in Grimsely et al, 2012), or else the conclusion that there are L4 vs. L2/3 differences rests on an artifact of undersampling, and would not hold up.

- We thank the reviewer for this suggestion. The observation of the reviewer is correct that we have fewer call-responsive A1 L2/3 neurons in the 3 – 6 kHz range. This is a consequence of the vocalization stimuli we used – in new Figure 2, we now provide both the overall spectra as well as spectrograms of the stimuli used in this study. There is a noticeable lack of spectral energy in the 4 – 6 kHz range. This is because the fundamental frequencies of many of the calls used in our study were well below 2 kHz (purrs, rumbles, chuts, whines), so that their harmonics had energy below 4 kHz. The calls with higher fundamental frequencies near 3 kHz (chirps and wheeks) had their harmonics in the >6 kHz range. We could record from many vMGB and A1 L4 neurons with their BFs in the 4 – 6 kHz range likely because their bandwidths stretched into the lower or higher ranges where call energy was present. We likely did not encounter many call-responsive A1 L2/3 neurons in the 4- 6 kHz range because although they had similar bandwidths that stretched into the lower or higher frequency range, this was insufficient to capture some underlying broadband feature to which these neurons responded.
- We of course don’t believe that A1 L2/3 neurons with BFs in the 3-6 kHz range don’t exist. The critical point is that in this study, we are only discussing neurons that responded to at least one of our call stimuli, and given the stimulus set used it is possible that we were able to drive fewer neurons with a BF in that range.

2) One place where this problem could lead to a misinterpretation is shown in Fig 3A. The main difference between A1 L2/3 and L4 appears to be the more uniform distribution across category number of the former vs. the more peaked distribution towards more categories of the latter. But if the BFs of L2/3 neurons are missing a spectrally dense region of the vocalizations, then it could make sense that there are not as many neurons whose responses track those acoustics as well as in the L4 or vMGB populations. Analyzing the response categories as a function of how neurons are tuned might be one way to get at this, but filling in the distribution of BFs around 3-6 kHz would be best.

- Please see our explanation above – our stimulus set did not have spectral energy in the 4 – 6 kHz range that resulted in us encountering fewer A1 L2/3 units in this range. Following the reviewer’s suggestion, we made a concerted effort to record from more A1 L2/3 neurons in this range from a new animal.
- We have added 5 new neurons with BFs between 4.5 kHz and 6.5 kHz to the dataset. Again, we would like to emphasize that it is not that we did not encounter A1 L2/3 neurons in this frequency range at all – only that it was quite difficult to encounter neurons in this frequency range that also responded to one of the calls in our stimulus set. (please see new Figure 2 and updated Figure 1 in the revised manuscript).

3) Related to above, the authors make the claim that L2/3 differences cannot be attributed to low-level differences in tuning properties. However, they only use tuning bandwidth as a comparison, and Fig 1F seems to show that L2/3 tuning in the 6-12 kHz BF range, where many of the calls probably have spectral content, looks to be particularly narrow compared to L4. A more thorough comparison of tuning properties as a function of best frequency would be helpful.

- We thank the reviewer for this suggestion. We now plot the bandwidth as a function of best frequency in Fig. 1G. We performed an analysis of covariance (ANCOVA) with best frequency as a covariate. We found a significant effect of best frequency on bandwidth across all the processing stages. After accounting for this difference, the bandwidth of vMGB neurons was significantly broader than the other two stages, but the bandwidths of A1 L4 and A1 L2/3 neurons were not significantly different across the entire frequency range. (please see Fig. 1G in the revised manuscript).

One could also look at predicting the response to calls based on the tuning curve itself. Note though that even if it were the case that tuning per se is not explanatory of the responses for some neurons, that would be consistent with findings from Eli Nelken's lab many years ago looking at how some cat auditory cortex neurons respond unexpectedly to the spectral context around bird vocalizations.

- Please see our response to comment 5, below. In the revision, we provide extensive analysis where we use linear-nonlinear models to estimate the spectrotemporal receptive fields of these neurons and predict their responses to calls (see new Figures 5, 6, and 7; Pages 13 – 18 of main text).

4) Conceptually, the authors push the idea that there is an "abrupt" transition between the way L4 and L2/3 of A1 encode these vocalizations. This is a potentially novel point. However, while the authors make a reasonable case that there are differences, whether those differences should constitute an "abrupt" change in coding or not is subjective. They do not provide any sense of what a "gradual" change would look like, and whether there is a quantitative way to differentiate "abrupt" from "gradual." In fact, many of the distributions comparing L4 to L2/3 look different, and while some figures show vMGB is not different from L4 (Fig 3A and B), others do not show vMGB data (Fig 4) at all, ignore differences between vMGB and L4 (Fig 3D), or do not indicate whether apparent differences are significant or not (Fig 3G).

- As we mentioned in the original submission, a “gradual” model would predict that the distributions of A1 L4 selectivity parameters would lie somewhere in-between the vMGB and A1 L2/3 distributions. One way to judge this (as the reviewer does) is to look at overall trends of statistical significance between the three processing stages across all parameters, with a gradual model predicting significant differences between all three stages and an ‘abrupt’ model predicting no significant differences between vMGB and A1 L4. In the revision we have done so – in new Table 1 (pages 22-23), we summarize all relevant statistical tests that we ran on vMGB, A1 L4, and A1 L2/3 neurons, grouped into differences in basic tuning, selectivity parameters, and information theoretic parameters. Of course, there are a couple of cases where vMGB and A1 L4 are significantly different. But most tests do not show a significant difference between vMGB and A1 L4, and do show a significant difference between A1 L4 and A1 L2/3.
- While we believe that this does indicate an ‘abrupt’ transition, we are happy to let readers come to this conclusion themselves based on the data presented. Therefore, we have changed the title of our manuscript to “**A complex feature-based representation of vocalizations emerges in the superficial layers of primary auditory cortex**”, Throughout the manuscript, we have toned down our language about an “abrupt” transition.

5) The authors claim, "A1 L2/3 responses were contingent on the presence of specific spectrotemporal features, resulting in a highly sparse representation of calls." However, besides showing a sparse but higher probability response across repeated trials at specific points in the stimulus, the authors do not really provide much to support this claim. It would make the paper stronger to show this, but at this point it is speculative. Presumably, many of these calls have rhythmically repeated elements. What are the spectrotemporal elements that the L2/3 neurons are responding to and why don't they respond when those same elements occur elsewhere in the sound?

- We agree that the statements regarding the relationship between tuning properties of neurons and their call responses were not supported by the analyses in the original submission. In the revision, we now provide extensive new analyses, summarized in new figures 5, 6, and 7, characterizing the receptive fields of call-selective and non-selective neurons across processing stages. Briefly, we used an available software package (NEMS) to fit linear-nonlinear (LN) models to call-responsive neurons. We observed that while the model performed similarly across the processing stages during training, the model did not generalize well to a validation dataset particularly in A1 L2/3, demonstrating the difficulty of capturing these responses using LN models. In addition, we show several examples of frequency-selective receptive fields in L4 and feature-selective receptive fields in A1 L2/3. Critically, we show that STRF complexity is not different between vMGB and A1 L4, but is significantly higher in A1 L2/3. These new analyses support our original statement that while A1 L4 responses are likely driven by spectral content (given the restricted frequency-selectivity of their STRFs), A1 L2/3 neurons are contingent on the presence of specific spectrotemporal features. (Please see new Figures 5, 6, and 7; Pages 13 – 18 of main text).
- The question of whether there are additional mechanisms that are not captured by this linear-nonlinear framework that might explain why some neurons do not reliably respond for repetitions of features within calls is very much an open question that we cannot answer presently. Nonlinear contextual effects have been documented in auditory cortex (Williamson et al., 2016), for example, that could cause modulation of repeated response elements that cannot be captured by the linear-nonlinear models we used here.

6) The authors have described a creative way to automatically identify putative regions of the spiking responses. However, they should start first by comparing overall evoked firing rates in the different areas; that by itself may show the main difference between regions (or if not, then their case for focusing on kurtosis and sparseness is stronger).

- This was indeed our first-pass choice when we initially explored these data. For example, in a poster we presented at ARO 2019 (figure below), we used a metric that was based on overall firing rate. Using that metric, the median selectivity of A1 L2/3 neurons was 3 and the median selectivity of A1 L4 population was 6.

- However, the issue was that when we used overall evoked firing rate, we found that some reliable A1 L2/3 call responses were classified as unresponsive, because the few reliably occurring spikes in a small time window within the call duration were drowned out by the zero responses in most other time windows in the call duration. Feedback at the ARO 2019 poster session also suggested that a more conservative metric should account for such reliable responses. It is to capture these smaller windows of activity that we chose the more complex analyses presented here. We believe that counting activity that occurs even in small windows is a quite conservative metric of selectivity.
- As suggested, we have now included a statement of the results of the overall firing rate analysis, its results, and limitations in the main text of the manuscript. (Page 9, Lines 223-231).

Furthermore, there is some arbitrariness to how they arrive at their bounding boxes and how regions get merged together. The authors should test whether their conclusions change as they change their criterion. For example, the neuron in Fig 2B (right) has many bounding boxes in response to a Chut, while the one in Fig 2B (left) looks like it has a similar pattern of responses to a Chirp. Yet in the former, the response is broken into many bounding boxes, while they are all merged together for the latter. This seems arbitrary based on an experimenter's empirical algorithm rather than what the brain may care about. For low firing neurons such as the one in the middle of Fig 2C, there are responses to Other or Rumble calls that are not merged together under the current criterion, but might have been if the criteria were more permissive to capture the rest of what appears to be an excitatory response.

- When we adopted the automatic detection algorithm, we chose parameters that broadly matched the visual judgements of independent human observers over a sample of neurons chosen across all processing stages. We tested multiple parameters and chose ones that better matched human judgements. While some minor differences arose, these parameter choices did not change the fundamental conclusions of this manuscript.
- Specifically, the decision to merge or not merge response windows did not change the selectivity at all (as expected), and only resulted in marginal changes to the number of response windows and response length (please see figures below).
- Selectivity:

- Response lengths:

(continued on next page)

- Number of response windows:

- In addition, to complement the response-window analysis, we had also presented analysis based on overall trial-wise firing rates that did not depend on our identification of response windows (kurtosis). We observed results that were consistent with the response window analysis. In the revision, we have added a third metric that is also not dependent on our identification of response windows (activity fraction) that is also consistent with the other two metrics (revised Fig. 4E).
- We have now added a note to the Methods section stating that we have tested a range of response window parameters and that this did not affect our conclusions (Page 32, Lines 813 – 816).

7) The authors report spontaneous activity differences in the Discussion, noting a p-value of only 0.03. This is a result, and should be put into the results section along with a plot of the distributions of the spontaneous activity across areas, just as for Fig 3 and 4, especially since they make an argument in the Discussion comparing spontaneous activity to other species. The concern is that the spontaneous activity is actually quite variable in all these areas, and that is not being reflected in the examples of Fig 2.

- We have now plotted the distributions of spontaneous rates across the processing stages in revised Fig. 1E. The result is stated in Page 7, Lines 168-172 of the revised manuscript.

8) Methodologically, the authors calculate stimulus-specific information by filling in responses after the end of stimuli of varying lengths with simulated spontaneous firing. Why? This seems completely unnecessary if the goal is to understand the true nature of how neurons are conveying information about stimuli. If the actual firing after the end of a stimulus is spontaneous, then that should be present in the trial-by-trial firing already; if not, then the results from simulating firing do not reflect the reality. It is also unclear why time bins before the start of the stimulus are used.

- To clarify, we filled in with simulated spontaneous spikes only after 1 second of stimulus offset (now accurately stated in Methods – page 35, line 887). This was done because calls varied in length, and mutual information calculations required rectangular matrices. While the recordings are continuous, because we presented stimuli with a randomized inter-trial interval, the next stimulus could potentially start after 1s of the previous stimulus’ offset. To avoid this confound, we filled in the matrix with the ‘null’ or ‘no response’ condition, for which the spontaneous rate seemed more appropriate than just zeros.
- For analyses in Figure 8, we used 100 ms time bins overlapping with a 50 ms slide. We used time bins starting 50 ms before stimulus onset because the latter half of the 100-ms wide time bin could contain stimulus-driven spiking.

9) While the information analyses take into account suppressed portions of the response, the data being analyzed consists only of neurons whose firing has an excitatory component, based on having an evoked rate 6 standard errors from the mean spontaneous activity in one of its windows. Prior research in awake mice has shown the importance of neurons that are fully suppressed by vocalizations. Presumably such neurons exist in the guinea pig as well, and some estimate of how often they are encountered in L4 or L2/3 would be useful. The information theoretic analysis could also include such neurons to give a more complete view.

- We apologize for the misunderstanding. We used all call-responsive neurons for which we acquired data from at least 10 repetitions, regardless of whether the response was inhibitory or excitatory, for all the information theoretic analyses. The only analyses in which we imposed the 6 s.e.m criterion mentioned pertained to the response window analysis summarized in Fig. 4B.

10) The Discussion is generally written in a scholarly manner with good coverage of existing literature in the guinea pig and nonhuman primate. However, the authors ignore relevant studies in other rodent species, including rats and mice. Moreover, several points are overstated.

- The authors claim an advance in using an unanesthetized preparation to study vocalization processing, but this has been done for many years in rats and mice.

- The authors claim an advance in using an extensive set of conspecific calls, but again there are many papers that do so in rats, mice and gerbils.

- We did not intend to convey the impression that we provide an advance by using the unanesthetized preparation or by using an extensive stimulus set – certainly, unanesthesized recordings have been performed in non-human primates as well. We intended to point out that gaps in understanding have remained because individual studies have addressed one or few of these conditions (anesthesia, stimulus richness, single-unit activity, laminar specificity), but not others, leaving gaps in understanding. For example, the Grimsley et al., 2013 study uses a rich stimulus set and describes responses in many auditory areas, but is done in anesthetized animals and did not consider laminar differences.
- We have now reworded this both in the Introduction (Page 5, Line 109) and Discussion sections (Page 24, Lines 593 – 597).

- The authors reference work from the Portfors lab to argue that mice are an outlier in showing an over-representation of social vocalizations in subcortical stations in a way that is not consistent with pure tone tuning. That argument is overstated, especially given other work from the Lesica lab using dense recording methods showing that responses to calls are largely consistent with best frequencies of neurons, as with other species. Furthermore, other species besides mice also show "associative and behavioral functions" in the responses of auditory cortical neurons. Hence, aside from the fact that guinea pig vocalizations are lower frequency than mouse ultrasonic calls, there is no reason to claim that mice are an "outlier."

- We have now removed the paragraph that refers to mice being an ‘outlier’. In the revision, we discuss results from both the Portfors and Lesica labs along with results from other species (Page 23, Lines 570-576).

11) The authors make the bold claim that their results indicate that "critical transformations to sound representations occur at the A1 L4 → A1 L2/3 synapse." There is simply no evidence to support that. All the studies reported here are based on extracellular recordings broken down by depth, with layer inferred from a current source density analysis. There are no whole cell recordings, cross-correlation analyses of simultaneously recorded units, etc. that would be expected to claim anything about the specific quoted synapse. In fact, based on studies in other rodents (e.g. Barbour and Calloway, Journal of Neuroscience, 2008), L2/3 neurons receive input not only from L4, but also from L2/3 and L5. What gives rise to the different coding of L2/3 vs. L4 neurons thus need not happen within just one synapse, especially given that the sparse response windows often occur late in the stimulus. The author's intuition may well be correct, but here they provide no evidence in support

of that case.

- We agree and this was bad wording in hindsight. In fact, in the original manuscript, we had indeed discussed possible roles of recurrent connectivity and inhibition on this transformation. We have reworded this sentence to read “Our data thus identify that critical transformations to sound representations occur at the superficial layers of A1. These data set the stage…” (Page 28, Line 692)

Other points

- Fig 1F - the experiments are based on recordings from 5 animals. To give more confidence that the results are not dominated by just a few animals, the best frequency plots could be modified to show different symbols for different animals so that the reader can weigh how much any one animal contributes to the results.

- Fig. 1F of the revision now displays the relative fraction of neurons arising from each subject used.

- Because the L2/3 distribution of call-selectivity is more uniform across the number of call categories rather than being peaked at 1 or 2, there are just as many neurons in L2/3 that are NOT call-selective. The language throughout should be toned down so that this is clearer, rather than giving the impression that one only finds call-selective responses in L2/3.

- We have toned down the language throughout in the revision, referring to “a third of A1 L2/3 neurons” or “many A1 L2/3 neurons” to avoid conveying the impression that we are referring to all A1 L2/3 neurons.

- The authors state, "Surprisingly and contrary to our expectation, we found even lower MI levels over the population of A1 L2/3 neurons (Fig. 4A, red)." I am not clear why this should be considered surprising. It fits with the lower firing rate and sparseness of the activity in L2/3.

- We have reworded this sentence to state that the results were consistent with the generally observed trend of decreasing mean information levels as one ascends the auditory processing hierarchy.

- Fig 3E should show an example vMGB neuron as well

- This figure (Fig. 4C in the revision) now includes an example MGB neuron.

- Fig 4 should show analyses for vMGB as well

- We now show these analyses for MGB as well (Figure 8 in the revised manuscript).

- "Data shows" is incorrect - "data" is plural

- Thank you, we have corrected this (Page 18, line 455).

Finally, concerning the use of statistics, the K-S test is fine for making claims that distributions are different, but it can be overly sensitive and lead to false positives. The use of the FDR correction is a not-particularly-conservative way to account for that, but still does not provide a way to claim anything about whether there is an "abrupt" difference or not between distributions.

- We have replaced the KS-tests with Kruskal-Wallis tests followed by Dunn-Sidak post-hoc pairwise comparisons throughout the manuscript. We have retained the 2D KS-test for comparing the response window lengths and numbers, but employ the Bonferroni correction for multiple comparisons.
